# Comparison of deforestation and forest land use factors for malaria elimination in Myanmar

**DOI:** 10.1016/j.ijregi.2023.06.006

**Published:** 2023-07-06

**Authors:** Amanda Hoffman-Hall, Robin Puett, Julie A. Silva, Dong Chen, Allison Bredder, Varada Shevade, Zay Yar Han, Kay Thwe Han, Poe Poe Aung, Christopher V. Plowe, Myaing M. Nyunt, Tatiana V. Loboda

**Affiliations:** a Eckerd College, Environmental Studies Discipline, St. Petersburg, USA; b University of Maryland, School of Public Health, College Park, USA; c University at Buffalo, Department of Geography, Buffalo, USA; d University of Maryland, Department of Geographical Sciences, College Park, USA; e Duke University, Global Health Institute, Durham, USA; f Department of Medical Research, Myanmar Ministry of Health and Sports, Yangon, Myanmar; g Malaria Consortium, Faculty of Tropical Medicine, Mahidol University, Bangkok, Thailand; h University of Maryland, School of Medicine, Baltimore, USA

**Keywords:** Malaria, Deforestation, Myanmar, LCLUC, Land cover, Land use

## Abstract

**Objectives:**

Within the remote region of Ann Township in Myanmar's Rakhine State, malaria prevalence has remained steady at ∼10% of the population from 2016-2019. Previous studies have linked areas of higher malaria prevalence in the region to heavily forested areas, however, little is known about how people live, work, and move through these areas. This work aims to disentangle landscape from land use in regard to malaria exposure.

**Methods:**

We investigated the roles of forest cover, forest loss, and land use activities with malaria prevalence through the combined use of land use surveys, malaria surveillance, and satellite earth observations.

**Results:**

Our results confirm previous research that linked areas of high forest cover with high malaria prevalence. However, areas experiencing high levels of deforestation were not associated with malaria prevalence. The land use factors that contribute most significantly to increased malaria risk remained those which put people in direct contact with forests, including conducting forest chores, having an outdoor job, and having a primary occupation in the logging and/or plantation industry.

**Conclusion:**

Malaria prevention methods in Myanmar should focus on anyone who lives near forests or engages in land use activities that bring them within proximity of forested landscapes, whether through occupation or chores.

## Highlights

• Deforestation in Myanmar is not linked to increased malaria incidence.
• Forest land use (plantation work, logging, gathering firewood, etc.) is linked to malaria.
• Malaria prevention methods should target those living or working near forests.

## Introduction

Affecting over 200 million people a year, the life-threatening vector-borne disease, malaria, remains a global health crisis [1]. However, many regions have reached low-transmission status through malaria control and elimination efforts supported by large funding increases over the last two decades. A significant success story is found in the Southeast Asian country of Myanmar, formerly known as Burma. Since peaking in 2010, Myanmar has reduced its number of malaria cases by a monumental 95% [1]. However, Myanmar continues to carry a heavy malaria burden, accounting for the majority (71%) of *Plasmodium falciparum* cases identified in the Greater Mekong Subregion in 2020 [1]. Further complicating matters, the remaining malaria transmission foci in Myanmar are heterogeneous and complex, and many remaining infections are clinically silent, rendering them invisible to routine monitoring [2], [3], [4], [5]. Therefore, targeted treatment and prevention strategies have been implemented across Myanmar to eliminate these remaining reservoirs.

The two principal vector control measures for malaria prevention implemented under the National Plan for Malaria Elimination in Myanmar focus on prevention from within the home [6]: Universal population coverage and use of long-lasting insecticidal nets (LLINs), and under some conditions, indoor residual spraying. While these measures have likely contributed to the 95% decrease in malaria cases across the country, within the remote region of Ann Township, Rakhine State, Myanmar, malaria prevalence has remained at nearly 10% of the population from 2016 [3] to 2019 (data presented here). Ann is highly rural and undeveloped, with forest areas covering nearly 70% of the township [7].

It is reasonable to assume that these home-centric strategies may not effectively eliminate this remaining malaria reservoir within Ann. However, very little research has considered how the people of Ann Township live, work and move through the landscape outside of their homes. Previous research has found village-level associations between malaria prevalence and the environment, specifically natural forest-dominated landscapes [3,8,9]. However, removing this landcover type has also been shown to increase malaria prevalence, with multiple studies observing increases in malaria alongside increases in deforestation [10], [11], [12].

The present study aimed to disentangle landscape from land use in regard to forest-related malaria exposure. We tested the associations between landcover type, landcover change, and land use with malaria incidence for the people of Ann Township, Myanmar.

## Methods

### Study site

**Figure 1:**
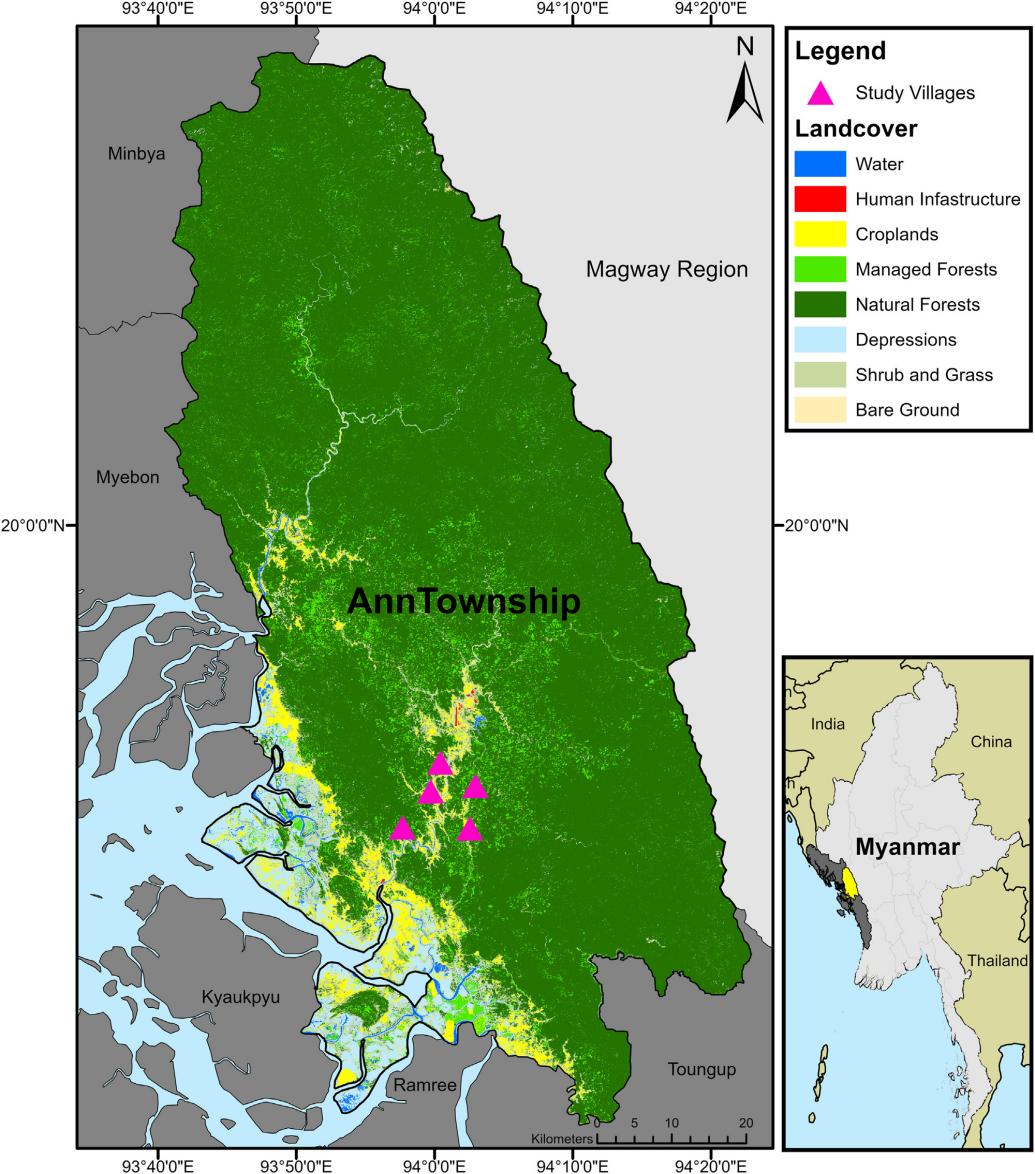
Study villages. Surveyed villages (offset and unlabeled to preserve privacy) overlaid a landcover map [13] of Ann Township, Rakhine State, Myanmar.

Five remote villages in southwestern Ann Township were surveyed (Figure 1). Ann has a subtropical climate, with a distinct monsoon season that stretches from April through October. The population of Ann is generally isolated, with highly uneven patterns of settlements covering less than 0.1% of the total land area [14]. Previous research has shown that isolated settlements experience disproportionate shares of adverse health outcomes [15] and can serve as the main drivers of infectious disease transmission into previously disease-free regions [16].

Ann Township is primarily dominated by two malaria vectors, forest-dwelling *Anopheles dirus,* and foothill and valley-dwelling *Anopheles minimus* [17]. Commonly identified malaria parasites include *Plasmodium vivax* and *P. falciparum,* but *P. knowlesi* has been identified elsewhere in Myanmar [13,18], though was not tested for in this study. For Ann specifically, the majority of infections are subclinical, meaning that infected persons show little to no symptoms, and the parasites can only be detected through ultrasensitive laboratory techniques as opposed to in-field rapid diagnostic testing (RDT) [3].

### Study population selection and data collection

This study employed a nested case-control study design, which analyzes a geographic subset of participants from a larger longitudinal study [19]. The full protocol can be found at www.clinicaltrials.gov, Identifier: NCT03483571. The study was independently reviewed and approved by the Institutional Review Boards of the Myanmar Department of Medical Research and Duke University. The village selection was based on the known or suspected malaria burden and research team's capability to ensure the integrity of study data and samples.

Surveying across five Ann Township villages was conducted from August 2018 through February 2019, in both the rainy and dry seasons. Village A was surveyed only during the rainy season; villages B and C were split between the rainy and dry seasons; villages D and E were surveyed during the dry season. The sampling framework was neither random nor fully representative of every village. However, unbiased prevalence estimates of *P. falciparum* and *P. vivax* infection were collected for villages A, B, and E, where all eligible villagers were sampled.

### Outcome: individual-level malaria prevalence

Analysis of the nested case-control data is conducted at the individual level, not the village level, since unbiased village prevalence does not exist for every village. Cases were those participants who tested positive for any malaria (*P. vivax* and/or *P. falciparum*) through ultrasensitive polymerase chain reaction methods [20], while controls were those that tested negative. A total of 1000 participants were enrolled and completed the study successfully for use in the nested case-control study.

The characteristics of the study population, study villages, and village-based malaria prevalence for *P. falciparum* mono-infection, *P. vivax* mono-infection, and mixed infection are summarized in Table 1.

**Table 1:** Area in square km (% of total area ∼12.56 square km) of proportion of natural forest cover loss within years preceding data collection and model results expressing the risk of *Plasmodium* presence in the blood as a function of forest loss. Blue cells indicate protective associations; red cells indicate risk associations; white cells indicate nonsignificant associations.

### Exposure: village-level natural forest cover

Hoffman-Hall *et al.* [3] show that the area of natural forest cover surrounding five different villages within Ann Township was highly associated with an increase in malaria risk, even for villagers who did not report visiting the forest frequently. The first priority of this study was to test if an association was also present between the villages in this study and their respective levels of natural forest coverage, and if so, control for natural forest cover in the statistical modeling to allow for the isolation of individual land use-associated risks. The area of natural forest cover was quantified based on a 30 m landcover map of Ann Township circa 2016 derived from satellite earth observation datasets [21]. The accuracy of the natural forest class for the map is 90%.

An area with a 2 km radius around each village was used to calculate the total area of proximal natural forest, following the estimated flight range of the major malaria vectors in the region - *An. minimus* and *An. Dirus* - 1 km and 2 km, respectively [22,23]. The 2016 natural forest class was updated to account for likely changes in land cover composition by 2018 when the malaria surveys were collected. To update the extent of proximal natural forest cover, Global Forest Change (GFC) [24] data from 2017 and 2018 was used to remove any areas of natural forest which were deforested between 2016 and the start of 2019.

### Exposure: village-level forest cover loss

Forest cover loss has been shown to increase malaria prevalence, with multiple studies observing increases in malaria alongside increases in deforestation in Indonesia and the Amazon [10], [11], [12]. To test if this association holds for Myanmar, we calculated the area of forest loss within 2 km of each village for the years between 2014 and 2018, derived from the GFC dataset. The annual rate of forest loss for the previous 5 years (2014-2018) and the total area of forest loss within that period were added as metrics of landscape-level proximal forest loss to the analysis. Shorter periods were also analyzed.

### Exposure: individual-level land use

Participants were surveyed on their participation in four land use activities within Ann Township: (1) attending to crops/farming; (2) working at plantations; (3) conducting household chores that involve trips to the water; and (4) conducting household chores that involve trips to the forest (e.g., hunting, firewood, and construction material collection, fruit gathering). Respondents were instructed to answer “Yes” if they had participated in that activity within the past 3 months. Each participant was also asked to identify their primary occupation and report if that occupation was primarily indoor or outdoor. The options provided for occupation were: dependent, student, vendor, soldier, refugee, farmer, plantation worker, mineworker, logger, and other (specify).

### Potential confounders and effect modifiers

Demographic and other relevant risk factor information was collected using the questionnaire, which included a range of questions based on malaria literature which commonly identifies the following variables as potential confounders: age [25], gender (self-identified) [26], pregnancy status [27], resident status (“have you lived in this village for >6 months?”) [28], and the seasonality of the participant's occupation (“does your main occupation vary seasonally in the past 1 year?”) [29]. Finally, each participant was surveyed about their bed net use, what type of bed net they used, and if they had slept under it the night before the survey.

### Statistical analysis

Univariate and multivariate logistic regression analysis was chosen to understand the association between the exposure variables and malaria prevalence. *P. falciparum, P. vivax*, and mixed infections were all considered as positive cases to conserve the validity of statistical testing with a small sample (total positive malaria cases were 9.60% of sample population, n = 96). Univariate analysis was performed initially to assess the relationship between each explanatory variable including proximal natural forest coverage, binary yes/no responses to the land use activities, primary occupation, and indoor/outdoor nature of that occupation with individual malaria infections.

For each univariate model where the assessed variable was found to be significant (*P* <0.05), potential confounders (age, age squared, gender, pregnancy status, resident status, bed net use, occupation seasonality, the season of data collection [rainy/dry]) were added progressively. Confounders found to be significant remained in the final adjusted model while nonsignificant variables were removed. Odds ratios (ORs) and 95% confidence intervals (CIs) were calculated for the resultant adjusted models. Finally, we conducted a sensitivity analysis that stratified participants by age groups (youth: 0-14 years old, and working-aged 15+) presented as supplementary material (Tables S4 and S5).

### Role of the funding source

The funders of the study had no role in study design, data collection, data analysis, data interpretation, the writing of the report, or in the decision to submit the paper for publication.

## Results

### Malaria prevalence and study population demographics

A total of 1000 participants completed the study (Table S1). Malaria prevalence across the villages was low overall. As shown in the supplementary material (Table S1), malaria was detected in 9.6% (n = 96) of the study population. *P. vivax* was the most prevalent, accounting for 53.1% (n = 51) of cases, in comparison to only 39.6% (n = 38) for *P. falciparum*. The remaining 7.3% of cases were mixed infections. The vast majority of cases were subclinical – only 5.2% (n = 5) of cases were detected by RDT.

Multiple covariates were determined to be significantly associated with malaria and therefore were included in the fully-adjusted models for our exposures of interest (natural forest cover, forest cover change, and land use participation). No effect modifiers were identified. The covariates included in the adjusted models are season of collection, age, age squared, gender, participant reporting conducting seasonal work, and use of a LLIN the night before the survey.

When sampled, participants were able to select multiple net types that they owned (ordinary, LLIN, or impregnated with insecticide). Ordinary nets were not found to be associated with malaria for this sample population. However, LLIN use the night before the survey was found to be strongly protective and, therefore, is included in all adjusted models. High compliance with LLIN use was found among the villagers, with 77.3% of participants sleeping under one the night before the survey (Table S1).

The sample population skewed toward women (self-identified) (52.8%) and young, with 35.7% of the sample under the age of 15 (Table S1 and S5). Only nine (0.9%) of the respondents indicated that they were pregnant at the time of data collection. Univariate analysis did not determine pregnancy to be a significant confounder. The sample population was made up primarily of permanent residents of the villages, with only six (0.6%) people reporting that they were not a resident of their village for the past 6 months. This was also not found to be a significant confounder. A little over a quarter of the study population reported having a seasonal job (27.1%) (“Does your main occupation vary seasonally in the past year?”), which was included as a confounder.

### Village-level natural forest cover results

**Figure 2:**
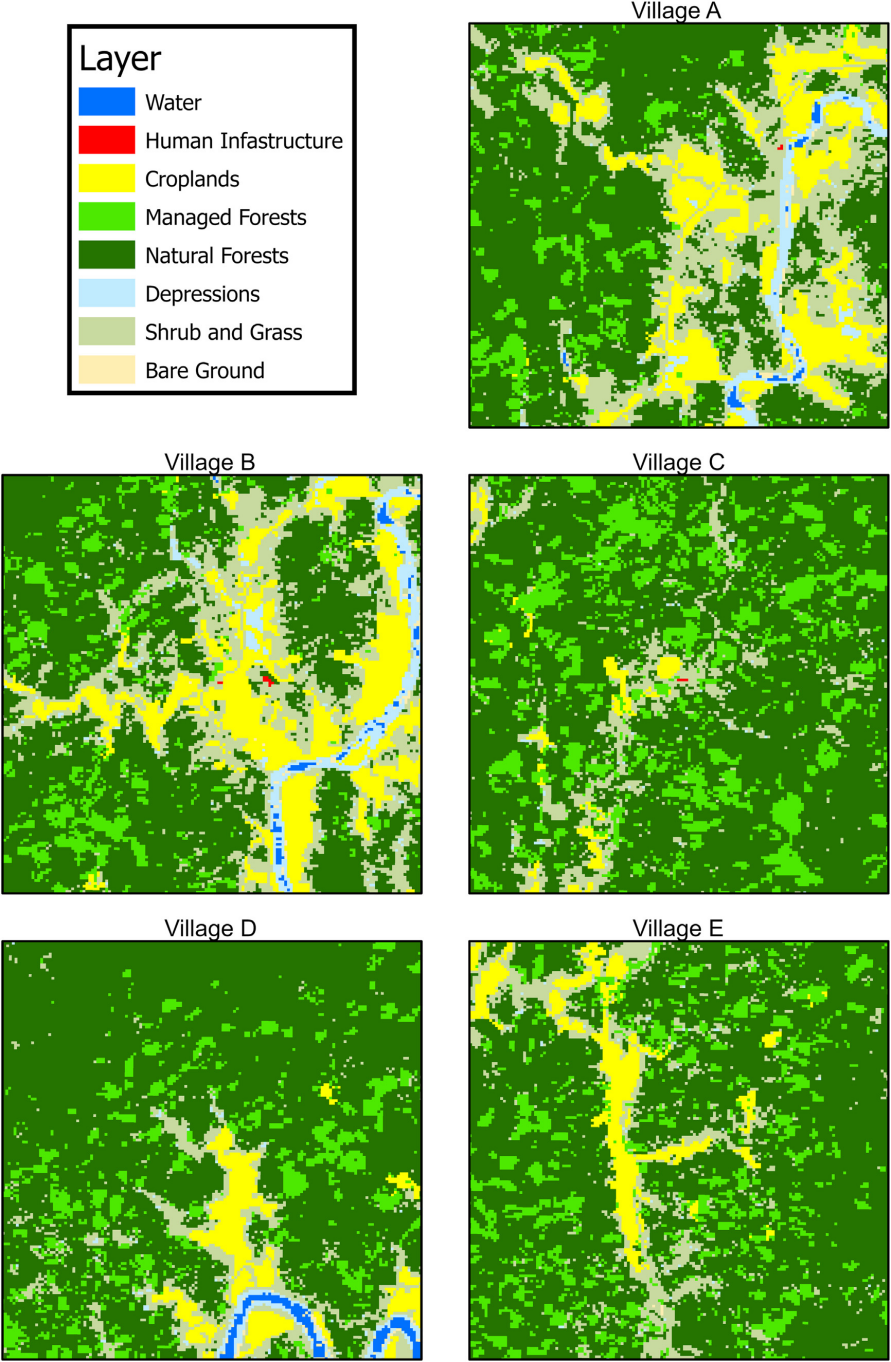
Land cover maps of the five surveyed villages. Exact locations not displayed for anonymity.

The land cover distribution around each of the villages varied widely (Figure 2), especially for the primary land cover of interest, natural forest. The proportion of natural forest ranged from a minimum of 41.2% in village B to a maximum of 63.8% in village D (Table S2). Similar to previous findings in Ann Township [3] area of natural forest within a 2 km radius (the estimated flight range of the major malaria vectors in the region*)* of a respondent's home village was found to be significantly associated with increased malaria risk (OR: 1.35, 95% CI: 1.08-1.72).

### Village-level forest cover loss results

Each village experienced forest cover loss in the 5 years preceding data collection (Table 1). Village C experienced the greatest amount of change, losing 3.86 square km between 2014 and 2018. Fully-adjusted models were created to assess the relationship between forest loss (deforestation) and malaria. Despite marked differences in the amount of forest loss across villages (Table 1), the only metric tested which showed an association with malaria was forest loss in 2018 (square km), which was found to be protective (i.e., the more forest removed in 2018 within 2 km of a participant's village, the lower the risk of malaria to that participant) (Table 1).

### Individual-level reported land use habits results

The results of the survey indicate that land use practices vary across Ann, both spatially and demographically (Table S3). The patterns of reported primary occupations within the surveyed villages closely mirror the landscape. For example, village C reports the highest proportion of forest workers (Table S3), which is aligned with village C also reporting the highest area of managed forests (Table S2) and the highest amount of forest lost from 2014-2018 (Table 1). Similarly, Village B claims the highest proportion of farmers (Table S3) and the highest proportion of croplands (Table S2). Village B was also the only village with a higher proportion of farmers than forest workers.

When conducting analyses of land use habits, proportion of natural forest cover surrounding a participant's village was added to the fully-adjusted models to control for the association identified between natural forest cover and malaria. As shown in Table S4, working in an outdoor occupation was found to be strongly associated with malaria (OR: 2.22, 95% CI: 1.10-4.65). In terms of reported primary occupation, a protective relationship was found for dependents (OR: 0.25, 95% CI: 0.09-0.57), while a strong association was found between malaria and forest-based occupations (i.e., loggers and plantation workers) (OR: 1.87, 95% CI: 1.12-3.16). Primary occupations of student, farmer, or other were not found to be associated with malaria (Table 2).

**Table 2:** Model results expressing the risk of *Plasmodium* presence in the blood as a function of reported land use. Blue cells indicate protective associations; red cells indicate risk associations; white cells indicate nonsignificant associations.

**Figure 3:**
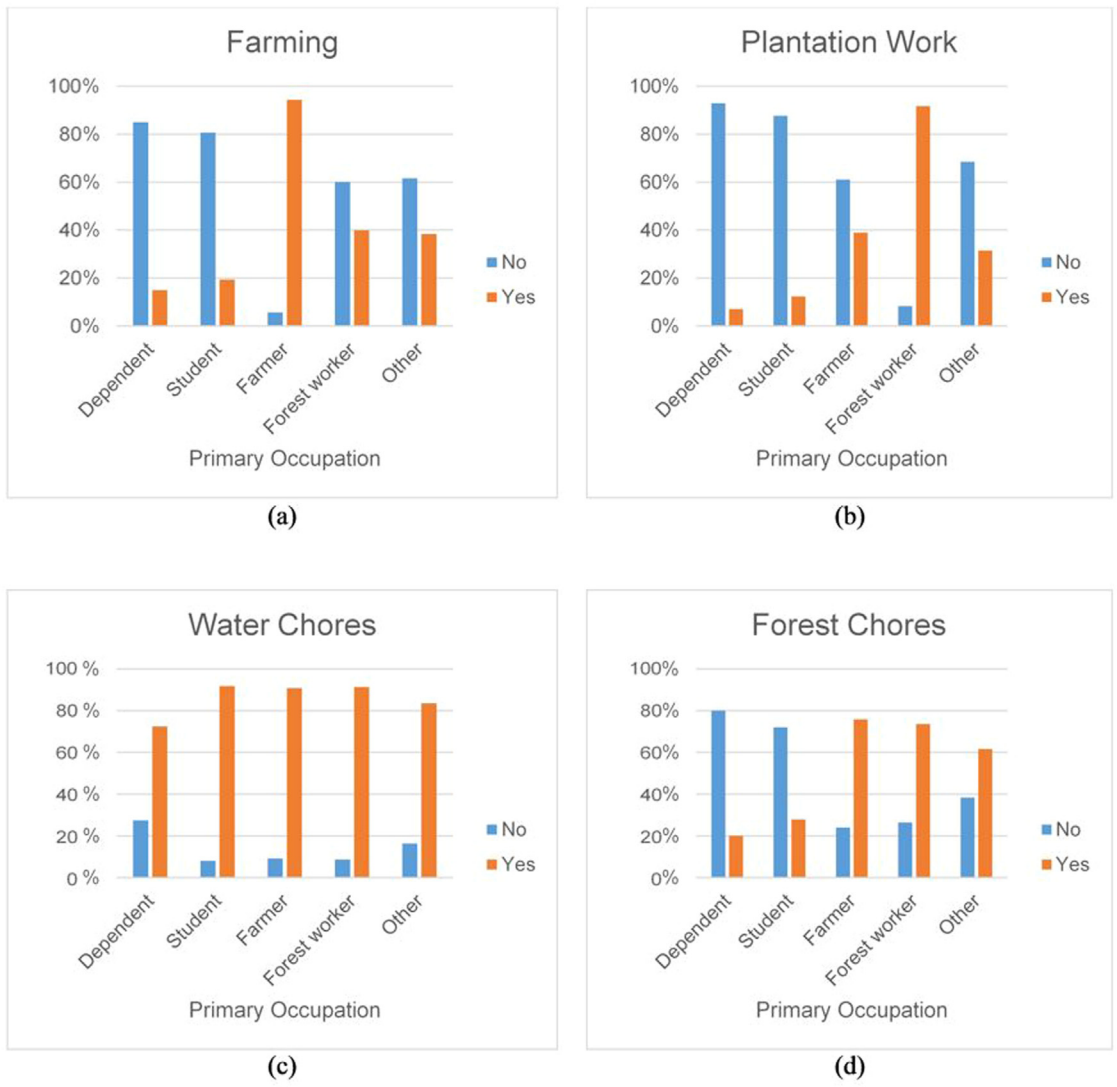
The proportion of surveyed villagers’ land use engagement based on their primary reported occupation. The proportion of the total surveyed village population per occupation is shown on the y-axis.

A critical finding is that reported primary occupation does not directly translate to land use activity engagement, which has many implications for targeted prevention strategies. For example, nearly 40% of the respondents who chose farmer as their primary occupation indicated that they also participate in plantation work (Figure 3b). Additionally, a small proportion of respondents who chose farmer as their primary occupation did not report engaging in any farming activities within the 3 months before the survey (Figure 3a). Similarly, a small but not insignificant group of students and dependents report engaging in farming and/or plantation work (Figures 3a and 3b). Since primary occupation fails to capture the range of land use activities that Ann residents engage in, fully-adjusted (including natural forest cover) models were created for each of the more specific land use options (Table 2). Reported engagement with plantation work was not found to be associated with malaria (OR: 1.58, 95% CI: 0.95-2.64), however engaging in forest chores (OR: 2.13, 95% CI: 1.27-3.66) was found to be strongly associated with malaria. No significant association was found between farming/attending crops or engaging in water chores (Table 2).

## Discussion

Malaria prevalence in Ann Township remains low; however, the overall prevalence in this study (9.6%) is very similar to the overall prevalence (9.4%) found in a similar population in 2016 [3]. While direct comparison is impossible due to village differences, it is reasonable to assume that malaria prevalence in Ann has not declined significantly from 2016-2019. The vast majority of cases in both studies were also subclinical, which may explain the lack of progress in malaria elimination. Moreover, these results indicate that the currently deployed strategy for malaria elimination is not sufficiently effective to achieve the goal of malaria elimination by 2030. Thus, new targeted interventions that can identify and treat the most likely carriers of subclinical malaria are of crucial importance to sustain the progress in malaria elimination achieved to date.

A major finding of this work is the wide diversity of land use activities undertaken by village residents, which is not fully represented by their primary occupation title. Malaria intervention strategies that target prevention toward specific occupations are likely less effective than intended. For example, a strategy that targets loggers for prophylactic measures due to their high level of interaction with the forest would miss the opportunity to prevent malaria in students aged 0-14 that also experience a high level of interaction with the forest through conducting forest chores.

Previous research has explored the link between forest workers and increased malaria risk [30,31]. However, to our knowledge, no previous study has controlled for village-level natural forest coverage of the workers’ villages through the use of satellite earth observations. The ability to efficiently and cost-effectively map the land cover surrounding our sampled villages was key to controlling for this variable within our modeling framework, which likely would have greatly influenced the results on land use exposure otherwise. This is supported by village D, which reported the highest prevalence of malaria (Table S1) and the highest amount of natural forest cover (Table S2). However, with only five villages, village-level analysis exhibits less statistical power than individual-level analysis.

Nevertheless, at the individual level, when the fractional natural forest cover surrounding a participant's village was controlled for, we found evidence of strong associations between forest-based primary occupations and malaria. This association was also seen in individuals who participate in forest chores. The group least at risk for malaria were those who claimed to be dependents, who were also the most likely to report little to no engagement with most of the land use activities (farming, plantation work, forest chores). This points to the efficacy of the current malaria prevention strategy in Myanmar, which focuses on vector control within the home. The World Health Organization has begun to call for prevention outside this domain, stating that “tools are also needed for the protection of people when they are outside of homes protected by core interventions owing to occupational or other reasons” in their Global Technical Strategy for Malaria 2016-2030. The results presented here indicate that this should be a priority for this region.

Following the high risk of malaria associated with natural forest landscapes that we identified, it seems counter-intuitive that a large body of research has observed a link between the removal of forests (deforestation) and malaria risk. We sought to answer if this relationship between deforestation and malaria is observable at the local scale in Ann Township, and, if so, is this relationship due to a shift in the ecology of the affected region (i.e., differences in vector preference for a natural vs. cleared forest as has been observed in Vietnam [5]), or instead because of activity by individuals within forested areas to clear the land. We did not find any association between the amount of local deforested land and malaria, with the notable exception of a strong decrease in risk of malaria for villagers living in an area with high amounts of deforestation within the year of data collection (2018).

We hypothesize that these results can be potentially explained by two mechanisms, (1) the future land use of a deforested area likely differs greatly in vector abundance, and (2) the link between malaria and deforestation operates on a larger scale than considered here in our focus on local, targeted interventions. In terms of future land use, previous research has found that in Ann Township forested landscapes allow for higher malaria exposure than croplands [3]. Therefore, the conversion of forests to croplands seems to lower the risk of malaria in that area. However, a high amount of forest conversion occurring in Ann Township is the clearing of natural forest for conversion to plantation (managed forest). We found that claiming a primary occupation that was forest-based (a category dominated by plantation workers) was associated with malaria, a finding also confirmed by studies conducted elsewhere in Myanmar [32,33]. A likely explanation for these results is that the vector ecology changes with landcover. A study along the China-Myanmar border (over 600 km from Ann Township) found that the pupation rate of *An. minimus* increased from 3.8% in natural forests (tropical rainforest) to 12.5% in banana plantations, to a substantially higher 52.5% in deforested areas [34]. To our knowledge, no such study exists for the natural bamboo and mixed deciduous forests of Ann, nor the rubber and teak plantations that dominate, though it is highly likely that these factors greatly influence vector abundance based on available evidence. The China-Myanmar border study also alludes to a temporal relationship (i.e., the transition from natural to cleared to plantation, with associated increases then decreases in vectors). A study that quantifies vector species abundance along the continuum of forest, to clear, to cropland, or plantation would be a welcome addition to the literature. It is possible that our null finding for deforestation may be influenced by the protective features of croplands.

Some limitations to the deforestation analysis presented here are that our village sample size is small, and the GFC data used is only available in a yearly format, which does not allow for the investigation of finer temporal scales. For example, while some of the respondent information was collected in early 2018, it is not possible to quantify how much forest loss occurred both before and after data collection. We also only analyzed forest loss per year and a single-year land cover map, not forest transition, which would be a welcome area for future work. As the economy of Myanmar grows, likely, the conversion of natural forests to rubber, teak, or other plantations will accelerate. Understanding the relationship between malaria and forest conversion will be critical to eliminating malaria under these rapidly changing socio-economic conditions.

Another explanation is that of scale. During a time of rapid environmental change in the Amazon region, de Castro *et al.* [35] defined “frontier malaria” as comprised of three spatial scales, individual, community, and state/national, alongside a temporal element, with different factors influencing malaria risk at each scale. In this study, our goal was to recommend targeted interventions (individual through community scale) due to the overall low-transmission rate of malaria within the region. Therefore, for each village, we only considered deforestation within a 2 km buffer around each village, consistent with the flight range of forest-dwelling *An. dirus.* However, in much of the literature that has noted increases in malaria alongside increases in deforestation, the scale at which this relationship was found was much larger. For example, in the Brazilian Amazon, Bauch *et al.* [36], Terrazas *et al.* [37], and Santos and Almeida [12] all consider malaria and deforestation rates at the municipal scale – more similar to an analysis that may have compared Ann Township to other townships, as opposed to our village-level analysis. In southeast Asia, Garg's [10] work in Indonesia is similar to our study in that they analyze village-level malaria data, however in order to consider forest type, they necessarily used coarse-scale (250 m) forest change data and consider the entire country of Indonesia. Each of the previous works recommends broad state-level intervention policies, such as increasing forest conservation programs [10,36]. Therefore, when our results and previous evidence are considered together, we believe that this work makes an important contribution at the individual-to-community scale, while not necessarily invalidating work completed at larger scales. For example, a country with limited antimalarial intervention capacity may use deforestation as a metric to help determine which municipalities or townships have the highest need. It may not be appropriate, however, for that country to use deforestation as a metric to determine which villages or individuals exhibit the highest need. Simply living near areas of high deforestation did not influence malaria risk in our study. Our results show that considering the specific land use activities of individuals when targeting interventions would be more appropriate.

## Conclusion

This research yielded a significant finding of the relationship between natural forest cover and malaria, and the absence of any risk relationship between local deforestation and malaria. This second finding appears at first to contradict other studies which have observed increased malaria risk alongside increased deforestation at larger scales, however, when natural forest cover surrounding a village is controlled for, the land use factors that contribute most significantly to increased malaria risk at the individual level are those which put people in direct contact with forests, including conducting forest chores, having an outdoor job, and having a primary occupation in the logging and/or plantation industry. This suggests that the previously observed relationship between deforestation and malaria is scale-dependent and likely related to the increased interaction between individual people and forested landscapes (i.e., entering a natural forest to clear it), a relationship that supports further investigation.

While land use practices varied widely within the study population, one theme emerged clearly. The current reservoir of malaria remaining in Ann Township is held by people who are exposed to malaria through their land use behaviors outside of their homes. While preventing exposure in the home may have directly influenced Myanmar's achievement of low-transmission status, now is the time to shift the strategies away from home in Ann and similar ecological and epidemiological settings where outdoor-biting vectors predominate. Prevention methods should focus on anyone who engages in land use activities that bring them within proximity of forested landscapes, even if those people claim an occupation that is not forest-related.

## Declarations of Competing Interest

The authors have no competing interests to declare.

## Appendix Supplementary materials

Image, application 1

Image, application 2

Image, application 3

Image, application 4

Image, application 5

Image, application 6
