# Supplementary material for: Comparison of deforestation and forest land use factors for malaria elimination in Myanmar
Source: IJID Reg. 2023 Jul 6;8:75–83. doi: 10.1016/j.ijregi.2023.06.006 (PMC10393544; doi:10.1016/j.ijregi.2023.06.006)
Supplement: Supplementary file 1 [file mmc1.docx]

**Supplementary Material**

*Sensitivity Analysis*

There were many observed differences in the land use habits of youth as compared to 15+ aged participants, therefore the fully-adjusted models were stratified by age to more fully explain the relationships found. The modeling results indicate that the exposure metrics which increase the likelihood of having malaria are different for youth as compared to 15+ aged participants (Table S1). While the associations between malaria and outdoor occupations, dependents, and forest-based occupations remain strong for the working-aged cohort, conducting forest chores appears not to be significantly associated with malaria for the working-aged cohort (OR: 1.82, 95% CI: 0.97 – 3.59) but is strongly associated for the youth cohort (OR: 2.67, 95% CI: 1.10 – 6.52). When the working-age cohort was considered alone, working on a plantation was found to be associated with malaria as well (OR: 1.92, 95% CI: 1.05 – 3.61).

A demographic comparison of youth and working-aged cohorts is provided in Table S2. Very few participants (1.2%, Table 6) in the youth subsample reported primary occupations that were not Dependent or Student. However, youth respondents did report engaging in different land use activities (17.9%, 12.0%, and 23.3% for Farming, Plantation Work, and Forest Chores, respectively), though at much lower rates than the 15+ population (46.3%, 54.1%, and 62.4% for Farming, Plantation Work, and Forest Chores, respectively) (Table S2).
