# Supplementary material for: Comparison of deforestation and forest land use factors for malaria elimination in Myanmar
Source: IJID Reg. 2023 Jul 6;8:75–83. doi: 10.1016/j.ijregi.2023.06.006 (PMC10393544; doi:10.1016/j.ijregi.2023.06.006)
Supplement: Supplementary file 2 [file mmc2.docx]

**Supplementary Material**

**Table S1** Descriptive statistics of the sample population

.

|  |  | Village | A | B | C | D | E | Total |
| --- | --- | --- | --- | --- | --- | --- | --- | --- |
| Population  Sampled |  | **n** | 185 | 345 | 190 | 200 | 80 | 1000 |
| Malaria+ | ***P. falciparum* mono** | **n (% of village total)** | 5 (2.7%) | 16 (4.6%) | 7 (3.7%) | 7 (3.5%) | 3 (3.8%) | 38 (3.8%) |
|  | ***P. vivax* mono** |  | 13 (7.0%) | 11 (3.2%) | 9 (4.7%) | 15 (7.5%) | 3 (3.8%) | 51 (5.1%) |
|  | **Mixed *P. falciparum* & *P. vivax*** |  | 1 (0.5%) | 2 (0.6%) | 0 (0.0%) | 4 (2.0%) | 0 (0.0%) | 7 (0.7%) |
|  | **Any malaria** |  | 19 (10.3%) | 29 (8.4%) | 16 (8.4%) | 26 (13.0%) | 6 (7.5%) | 96 (9.6%) |
| Gender | **Women** | **n (% of village total)** | 87 (47.0%) | 183 (53.0%) | 106 (55.8%) | 112 (56.0%) | 40 (50.0%) | 528 (52.8%) |
| Age |  | **Years (Mean ± SD)** | 25.1 ± 18.6 | 30.0 ± 21.3 | 30.5 ± 15.8 | 25.4 ± 19.3 | 18.2 ± 15.8 | 27.3 ± 19.4 |
| Seasonal  Occupation | **Yes** | **n (% of village sample total)** | 31 (16.8%) | 117 (33.9%) | 55 (28.9%) | 55 (27.5%) | 13 (16.3%) | 271 (27.1%) |
| Slept Under LLIN Night Before Survey | **Yes** | **n (% of village sample total)** | 157 (84.9%) | 230 (66.7%) | 178 (93.7%) | 138 (69.0%) | 70 (87.5%) | 773 (77.3%) |
