# Supplementary material for: Comparison of deforestation and forest land use factors for malaria elimination in Myanmar
Source: IJID Reg. 2023 Jul 6;8:75–83. doi: 10.1016/j.ijregi.2023.06.006 (PMC10393544; doi:10.1016/j.ijregi.2023.06.006)
Supplement: Supplementary file 3 [file mmc3.docx]

**Supplementary Material**

**Table S2** Area in sq km (% of total area ~12.56 sq km) of each land cover type found within a 2 km radius of the village center.

|  | Village | A | B | C | D | E |
| --- | --- | --- | --- | --- | --- | --- |
| Population Sampled | **n** | 185 | 345 | 190 | 200 | 80 |
| Malaria+ | **n (% of village sample total)** | 19 (10.3%) | 29 (8.4%) | 16 (8.4%) | 26 (13.0%) | 6 (7.5%) |
| Landcover | **Natural Forest** | 5.24 (41.8%) | 5.17 (41.2%) | 6.74 (53.7%) | 8.01 (63.8%) | 7.28 (58.0%) |
|  | **Managed Forest** | 1.76 (14.0%) | 1.49 (11.8%) | 4.37 (34.8%) | 2.56 (20.4%) | 3.11 (24.7%) |
|  | **Human Infrastructure** | 0.14 (1.1%) | 0.16 (1.3%) | 0.15 (1.2%) | 0.08 (0.7%) | 0.11 (0.9%) |
|  | **Croplands** | 2.01 (16.0%) | 2.46 (19.6%) | 0.24 (1.9%) | 0.76 (6.1%) | 0.87 (6.9%) |
|  | **Shrub and Grass** | 2.64 (21.1%) | 2.43 (19.4%) | 0.90 (7.1%) | 0.82 (6.5%) | 0.99 (7.9%) |
|  | **Water** | 0.47 (3.8%) | 0.50 (4.0%) | 0.04 (0.3%) | 0.20 (1.6%) | 0.05 (0.4%) |
|  | **Bare Surface** | 0.30 (2.4%) | 0.35 (2.7%) | 0.12 (1.0%) | 0.13 (1.0%) | 0.16 (1.3%) |
