# Supplementary material for: Comparison of deforestation and forest land use factors for malaria elimination in Myanmar
Source: IJID Reg. 2023 Jul 6;8:75–83. doi: 10.1016/j.ijregi.2023.06.006 (PMC10393544; doi:10.1016/j.ijregi.2023.06.006)
Supplement: Supplementary file 4 [file mmc4.docx]

**Supplementary Material**

**Table S3** Reported primary occupations, job locations, and engagement in land use activities across sampled villages

|  | Village |  | A | B | C | D | E | Total |
| --- | --- | --- | --- | --- | --- | --- | --- | --- |
| Occupation  Location | **Indoor** | **n (% of village total)** | 106 (57.3%) | 188 (54.5%) | 72 (37.9%) | 108 (54.0%) | 52 (65.0%) | 526 (52.6%) |
|  | **Outdoor** |  | 79 (42.7%) | 157 (45.5%) | 118 (62.1%) | 92 (46.0%) | 28 (35.0%) | 474 (47.4%) |
| Primary Occupation | **Dependent** | **n (% of village total)** | 40 (21.6%) | 111 (32.2%) | 21 (11.1%) | 43 (21.5%) | 25 (31.3%) | 240 (24.0%) |
|  | **Student** |  | 59 (32.0%) | 65 (18.8%) | 44 (23.2%) | 63 (31.5%) | 27 (33.8%) | 258 (25.8%) |
|  | **Farmer** |  | 16 (8.6%) | 76 (22.0%) | 19 (10.0%) | 25 (12.5%) | 5 (6.2%) | 141 (14.1%) |
|  | **Forest-Based**  **Occupation** |  | 57 (30.8%) | 66 (19.1%) | 83 (43.6%) | 59 (29.5%) | 23 (28.7%) | 288 (28.8%) |
|  | **Other** |  | 13 (7.0%) | 27 (7.8%) | 23 (12.1%) | 10 (5.0%) | 0 (0.0%) | 73 (7.3%) |
| Land Use Activities | **Attending to Crops/Farming** | **n (% of village total that responded “Yes” to engaging in activity within past 3 months)** | 33 (17.8%) | 147 (42.6%) | 71 (37.4%) | 90 (45.0%) | 21 (26.3%) | 362 (36.2%) |
|  | **Working on a**  **Plantation** |  | 63 (34.1%) | 110 (31.9%) | 106 (55.8%) | 66 (33.0%) | 46 (57.5%) | 391 (39.1%) |
|  | **Conducting household chores that involve trips to the water** |  | 138 (74.6%) | 285 (82.6%) | 171 (90.0%) | 198 (99.0%) | 71 (88.8%) | 863 (86.3%) |
|  | **Conduct** **household chores that involve trips to the forest** |  | 57 (30.8%) | 170 (49.3%) | 117 (61.6%) | 107 (53.5%) | 33 (41.3%) | 484 (48.4%) |
