# Supplementary material for: Comparison of deforestation and forest land use factors for malaria elimination in Myanmar
Source: IJID Reg. 2023 Jul 6;8:75–83. doi: 10.1016/j.ijregi.2023.06.006 (PMC10393544; doi:10.1016/j.ijregi.2023.06.006)
Supplement: Supplementary file 5 [file mmc5.docx]

**Supplementary Material**

**Table S4** Model results expressing the risk of Plasmodium presence in the blood as a function of reported land use. Blue cells indicate protective associations; red cells indicate risk associations; white cells indicate non-significant associations.

|  |  | Odds Ratio  (95% Confidence Interval) | Odds Ratio for Sensitivity  Analysis:  Youth (0-14) | Odds Ratio for Sensitivity  Analysis:  Working-Age (15+) |
| --- | --- | --- | --- | --- |
| Occupation Location | **Indoor** | 1.00 | 1.00 | 1.00 |
|  | **Outdoor** | 2.21 (1.10 – 4.65) | 2.96 (0.11 – 33.79) | 4.08 (1.60 – 12.70) |
| Primary  Occupation | **Dependent** | 0.25 (0.09 – 0.57) | 0.72 (0.12 – 2.84) | 0.21 (0.05 – 0.65) |
|  | **Student** | 1.87 (0.91 – 4.04) | 1.12 (0.32 – 5.09) | NA |
|  | **Farmer** | 0.86 (0.43 – 1.62) | NA | 0.88 (0.45 – 1.67) |
|  | **Forest-Based Occupation** | 1.87 (1.12 – 3.16) | NA | 2.06 (1.18 – 3.65) |
|  | **Other** | 0.81 (0.32 – 1.78) | NA | 0.93 (0.36 – 2.07) |
| Land Use | **Attending to Crops/Farming** | 0.77 (0.45 – 1.28) | 0.75 (0.20 – 2.20) | 0.78 (0.43 – 1.40) |
|  | **Working on a Plantation** | 1.58 (0.95 – 2.64) | 1.32 (0.35 – 4.05) | 1.92 (1.05 – 3.61) |
|  | **Conduct** **household chores that involve trips to the water** | 1.74 (0.81 - 4.30) | 0.56 (0.18 – 2.19) | 2.84 (0.98 – 12.05) |
|  | **Conduct** **household chores that involve trips to the forest** | 2.13 (1.27 - 3.66) | 2.67 (1.10 – 6.52) | 1.82 (0.97 – 3.59) |
