# Supplementary material for: Comparison of deforestation and forest land use factors for malaria elimination in Myanmar
Source: IJID Reg. 2023 Jul 6;8:75–83. doi: 10.1016/j.ijregi.2023.06.006 (PMC10393544; doi:10.1016/j.ijregi.2023.06.006)
Supplement: Supplementary file 6 [file mmc6.docx]

**Supplementary Material**

**Table S5** Differences between the sampled population for the primary and sensitivity analyses for land use activities analysis.

|  |  |  | Youth (0-14) | Working-Age (15+) |
| --- | --- | --- | --- | --- |
| Population  Sampled |  | **n** | 357 | 643 |
| Malaria+ | **Any malaria** | **n (% of population sample)** | 28 (7.8%) | 68 (10.6%) |
| Gender | **Women** | **n (% of population sample)** | 168 (47.1%) | 360 (56.0%) |
| Age |  | **Years (Mean ± SD)** | 8 **±** 3.8 | 38 **±** 15.9 |
| Occupation  Location | **Indoor** | **n (% of population sample)** | 352 (98.6%) | 174 (27.1%) |
|  | **Outdoor** |  | 5 (1.4%) | 469 (72.9%) |
| Primary  Occupation | **Dependent** | **n (% of population sample)** | 104 (29.1%) | 136 (21.2%) |
|  | **Student** |  | 248 (69.5%) | 10 (1.6%) |
|  | **Farmer** |  | 2 (0.6%) | 139 (21.6%) |
|  | **Forest-Based  Occupation** |  | 1 (0.3%) | 287 (44.6%) |
|  | **Other** |  | 1 (0.3%) | 62 (9.6%) |
| Land Use | **Attending to Crops/Farming** | **n (% of population sample that responded “Yes” to engaging in activity within past 3 months)** | 64 (17.9%) | 298 (46.3%) |
|  | **Working on a  Plantation** |  | 43 (12.0%) | 348 (54.1%) |
|  | **Conduct** **household chores that involve trips to the water** |  | 309 (86.6%) | 554 (86.2%) |
|  | **Conduct** **household chores that involve trips to the forest** |  | 83 (23.3%) | 401 (62.4%) |
